# Hot Deformation Behavior of Free-Al 2.43 wt.% Si Electrical Steel Strip Produced by Twin-Roll Strip Casting and Its Effect on Microstructure and Texture

**DOI:** 10.3390/ma17133152

**Published:** 2024-06-27

**Authors:** Huihui Wang, Wanlin Wang, Peisheng Lyu, Shengjie Wu

**Affiliations:** 1 School of Metallurgy and Environment, Central South University, Changsha 410083, China; tsingwang2018@163.com (H.W.);; 2 National Center for International Research of Clean Metallurgy, Central South University, Changsha 410083, China

**Keywords:** non-oriented electrical steel, hot deformation, recrystallization, strip casting, texture

## Abstract

Twin-roll strip casting (TRSC) technology has unique advantages in the production of non-oriented electrical steel. However, the hot deformation behavior of high-grade electrical steel produced by TRSC has hardly been reported. This work systematically studied the hot deformation behavior of free-Al 2.43 wt.% Si electrical steel strip produced by twin-roll strip casting. During the simulated hot rolling test, deformation reduction was set as 30%, and the ranges of deformation temperature and strain rate were 750~950 °C and 0.01~5 s^−1^, respectively. The obtained true stress–strain curves show that the peak true stress decreased with an increase in the deformation temperature and with a decrease in the strain rate. Then, the effect of hot deformation parameters on microstructure and texture was analyzed using optical microstructure observation, X-ray diffraction, and electron backscattered diffraction examination. In addition, based on the obtained true stress–strain curves of the strip cast during hot deformation, the constitutive equation for the studied silicon steel strip was established, from which it can be found that the deformation activation energy of the studied steel strip is 83.367 kJ/mol. Finally, the kinetics model of dynamic recrystallization for predicting the recrystallization volume percent was established and was verified by a hot rolling experiment conducted on a rolling mill.

## 1. Introduction

Currently, the demand for non-oriented silicon steel is increasing towards high magnetic induction, low iron loss, thin specification, and high strength [1,2]. Twin-roll strip casting (TRSC) technology can directly cast liquid steel into a thin strip of several millimeters of thickness, which can greatly reduce the difficulty of hot rolling. In addition, sub-rapid solidification happens during direct contact between the liquid steel and the roller [3], which offers several advantages compared to traditional casting technology, such as improved microstructure, favorable texture, and environmental friendliness [4]. Therefore, TRSC technology provides a promising approach to produce high-grade silicon steel with high performance and thin specifications [5,6]. However, practical challenges related to production and process control during strip casting still need to be addressed [7,8,9]. Hot rolling is an effective method to reduce thickness and modify the initial structure and texture of non-oriented silicon steel. Consequently, investigating the rheological behavior and texture evolution during hot deformation of non-oriented silicon steel is important in controlling recrystallization texture and optimizing properties.

Liu et al. [10] reported that a 6.5 wt.% Si-0.3 wt.% Al non-oriented electrical steel as-cast strip with strong columnar grains was produced by laboratory-scale twin-roll strip caster, and subsequently, the as-cast strip was treated with hot rolling, warm rolling, and annealing. It was shown that the warm rolled sheet was characterized by strong α-fiber (<110>∥RD) texture but weakened γ-fiber (<111>∥ND) texture and an inhomogeneous deformed microstructure. Xu et al. [11] successfully manufactured non-oriented electrical steel with ultra-high magnetic induction using a laboratory-scale twin-roll strip caster, and its microstructure and texture evolution were studied in detail. Song et al. [12] investigated the effect of reduction rate on microstructure and texture during the reheating and hot rolling of thin strip electrical steel produced by strip casting. Jiao et al. [13] introduced hot rolling after strip casting, effectively improved the microstructure, and increased the nucleation of {001} fiber texture when the reduction rate was increased from 25% to 55% at 1000 °C. The above studies have discussed the effect of temperature and reduction rate on the microstructure and texture of silicon steel produced by TRSC technology, but the effect of strain rate on its texture and recrystallization have hardly been reported.

During the hot deformation process of silicon steel, rolling parameters can be controlled to adjust the dynamic recrystallization (DRX) behavior, thereby improving the homogenization of the microstructure and forming a favorable texture in the final product [14,15,16]. The hot deformation behavior of 5.5~6.5 wt.% Si steels has been reported, and the hot activation energy is in the range of 310.425~422.162 kJ/mol [17]. The effect of the Zener–Hollomon (Z) parameter on the microstructure of Fe-3 wt.% Si prepared by the traditional casting process has been studied, and the hot deformation activation energy was estimated as 289 kJ/mol [18]. Atake et al. [19] reported the effect of temperature and strain rate on the stress and texture of ultra-low carbon silicon steel with 2.5 wt.% Si and discussed the evolution of shear bands during the hot deformation process. The study concluded that the shear bands were easily generated by rolling at a lower temperature and strain rate, which led to the formation of fine grains. In summary, it can be seen that the studies on the hot deformation behavior of silicon steel are mainly of the traditional casting process, but the hot deformation behavior of silicon steel produced by TRSC technology has hardly been reported.

The influence of temperature and strain rate on the microstructure and texture of electrical steel during the hot rolling of silicon steel produced by TRSC technology has been subjected to limited investigation. Specifically, there is an absence of models for rheological behavior and dynamic recrystallization. This work aims to clarify the hot deformation behavior of non-oriented silicon steel as-cast strip prepared by TRSC technology based on the true stress–strain curves obtained during the hot rolling simulation. Then, the effect of deformation parameters on microstructure and texture was analyzed through an optical microscope (OM), X-ray diffraction (XRD), and electron backscattered diffraction (EBSD). The constitutive equation for describing the relationship among temperature, stress rate, and strain rate of the studied silicon steel strip was established. In addition, the dynamic recrystallization kinetics were also established to study the DRX behavior during the hot deformation process of silicon steel as-cast strip. This work can provide theoretical guidance for controlling microstructure and texture during the hot rolling process of non-oriented electrical steel as-cast strip.

## 2. Experiments 

### 2.1. Experimental Procedure and Characterization Methods

**Figure 1:**
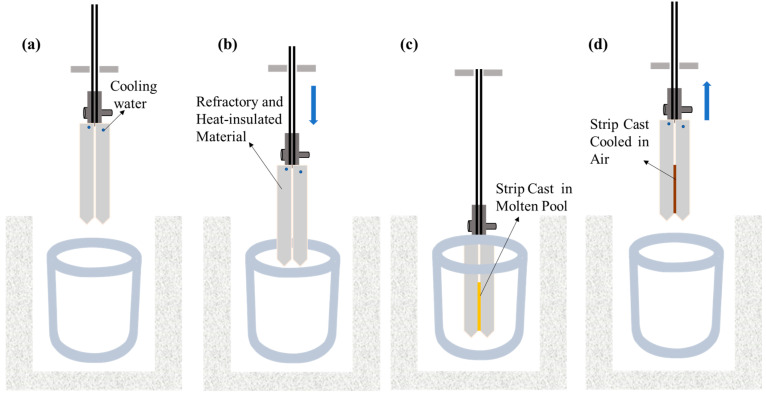
Experimental procedure of the fast dip tester: (**a**) molten steel at target temperature, (**b**) immersion of copper substrate, (**c**) staying for a certain time, and (**d**) withdrawal of copper substrate.

Non-oriented electrical steel with the major chemical composition of 0.0022C-2.43Si in wt.% was selected for this study; it is a medium-grade silicon steel. A fast dip tester was used in this study to simulate twin-roll strip casting and to obtain the strip cast of non-oriented electrical steel. The dip tester is used to simulate the process of twin-roll strip casting and has the advantages of convenient operation, low experimental cost, and of producing a cast strip microstructure that is close to that of industrial twin-roll strip casting. The detailed information of the dip tester can be seen in our previous study [20,21]. Figure 1 shows the experimental procedure of the fast dip tester. Firstly, about 5 kg studied steel after rust cleaning was placed into an induction heating furnace. When the molten steel reached the target temperature (1563~1568 °C (±10 °C), Figure 1a), the water-cooling copper substrate with two cooling faces was immersed in molten pool quickly (Figure 1b) and left for 0.35 s (Figure 1c) to simulate the contact condition between two copper rolls and molten steel of an actual strip caster. Then, the copper substrate was withdrawn quickly from the molten pool and cooled in air to form solidified strip cast (Figure 1d). The thickness of the obtained strip cast is 1.8~2.0 mm. 

**Figure 2:**
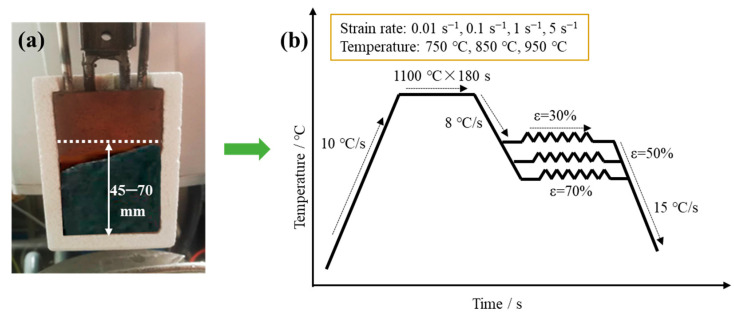
(**a**) The obtained strip cast, and (**b**) the processing schedule during hot rolling simulation test of silicon steel strip cast.

The strip cast obtained by the dip tester was cut into discs with a diameter of 6 mm along the thickness direction for the hot rolling simulation test, which was conducted on a thermo-mechanical simulator (Gleeble-3800) (Fule Test Technology Co. Ltd., Shanghai, China). To simulate the practical industrial process, all samples were first soaked at a rate of 10 °C/s to 1100 °C, then held for 180 s, and subsequently cooled to the deformation temperature at the cooling rate of 8 °C/s. The deformation temperatures were set as 750 °C (±20 °C), 850 °C (±20 °C), and 950 °C (±20 °C) with the strain rate of 0.1 s^−1^ and the reduction rate of 30%. The strain rates were set as 0.01 s^−1^, 0.1 s^−1^, 1 s^−1^, and 5 s^−1^, with the deformation temperature of 950 °C and the reduction rate of 30%. The deformed samples were then cooled to room temperature at the cooling rate of 15 °C/s. The processing schedule for the hot rolling simulation test is shown in Figure 2. Finally, the hot rolling experiment of the obtained as-cast strip was also conducted on the pilot rolling mill for verifying the dynamic recrystallization kinetics, and the reduction rates were set as 30%, 50%, and 70% with the hot rolling temperature of 750 °C and strain rate of 0.1 s^−1^. 

The samples were ground, polished, and then etched with 4% nital before the observation of optical microstructure by on optical microscope (OM, BX3M-LEDR) (Chongqing YongChang Technology Co. Ltd., Chongqing, China). Next, the grain size of the sample was determined by the area calibration method using Nano Measurer 1.2. In order to obtain the macro-texture, the {200}, {220}, and {211} pole figures from BRUKER X-ray Diffraction (XRD, DMAX-2500PC) (Shanghai Verde Instrument Technology Co. Ltd., Shanghai, China) were processed to obtain the orientation distribution function (ODF). The grain boundaries were characterized by ZEISS Scanning Electron Microscopy (SEM, JSM-7900F) (Zeiss, Shanghai, China) equipped with Electron Backscatter Diffraction (EBSD, JSM-7001F) (Zeiss, Shanghai, China) [22,23,24]. EBSD data were analyzed using OXFORD-HKL Channel 5.0.

### 2.2. Constitutive Equation for Hot Deformation

The Zener–Hollomon parameter plays a pivotal role in the hot deformation process, serving as a comprehensive indicator of deformation temperature, stress, and strain rate. In the traditional hot rolling process, the constitutive equation and Zener–Hollomon parameter are used to describe the hot deformation behavior of metal materials, but the research on the hot deformation behavior of silicon steel strip prepared by twin-roll strip casting is not widely reported. Therefore, the constitutive equation and Zener–Hollomon parameter are also used to study the two-roll thin strip non-oriented silicon steel. The Zener–Hollomon parameter is helpful in describing the high-temperature deformation behavior of metals. Specifically, the recrystallization is not easy to generate within certain Z values (lnZ = 28.44~28.62) [25,26]. The following equation can calculate the Zener–Hollomon parameter:

(1) $$Z=\dot{\varepsilon }\exp\left(\frac{Q}{\mathrm{RT}}\right)={\;A\left[\sin h\;\left(\alpha \sigma_{p}\right)\right]}^{n}$$

where $\dot{\varepsilon }$ is strain rate in s^−1^, *Q* is the deformation activation energy in kJ/mol, *R* is the gas constant and is equal to 8.314 J/(mol·K), *T* is the deformation temperature in °C, *σ_p_* is the peak stress in MPa, *A*, *α*, and *n* are material constants, among which α varies from 0.004 to 0.03 [27,28]. According to Equation (1), the relationship of strain rate with temperature and stress could be deduced as follows:

(2) $$\dot{\varepsilon }=A{[\sin h(\alpha \sigma_{p})]}^{n}\exp(\frac{-Q}{\mathrm{RT}})$$

## 3. Results and Discussion

### 3.1. True Stress–Strain Curves of Strip Cast during Hot Deformation

**Figure 3:**
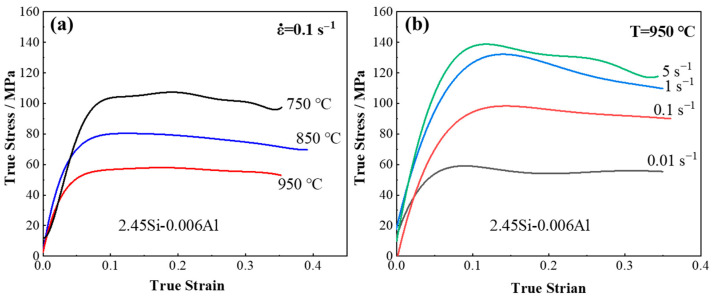
True stress–strain curves of the 2.43 wt.% Si steel strip obtained at (**a**) different deformation temperatures with strain rate 0.1 s^−1^ and (**b**) different strain rates with hot rolling temperature of 950 °C.

In order to investigate the effect of deformation temperature and strain rate on the mechanical properties of 2.43 wt.% Si steel strip cast samples, the true stress–strain curves during hot deformation are measured by Gleeble, and are shown in Figure 3. Figure 3a shows the true stress–strain curves obtained at different deformation temperatures with strain rate 0.1 s^−1^, where the peak stress decreases from 113 MPa at 750 °C to 58 MPa at 950 °C. Therefore, the flow stress decreases significantly with the increase in temperature. Figure 3b shows the true stress–strain curves obtained at different strain rates with hot rolling temperature of 950 °C, from which it can be found that the peak stress increases from 79 MPa of 0.01 s^−1^ to 151 MPa of 5 s^−1^. It is indicated that the flow stress values increase with the increase in strain rate. 

The true stress–strain curves shown in Figure 3 can be divided into two types. Work hardening is the main phenomenon during the early stage of deformation. At the low strain rate (0.01 s^−1^ and 0.1 s^−1^), the DRX behavior is significant. During work hardening, dynamic recovery (DRV) and DRX are in equilibrium, and the stress reaches its peak value and then stays steady with the increase in true strain. At the high strain rate (1~5 s^−1^), the DRV behavior is predominant. After the true stress reaches its peak value, it begins to decrease with the increase in true strain. This can be attributed to the smaller grain size at the low strain rate compared to the high strain rate, which is caused by the evident DRX under the low-strain-rate condition. The coarse grain size at the high strain rate is not conducive to the deformation resistance, and thus results in a reduction in deformation stress.

### 3.2. Effect of Deformation Parameters on Microstructure and Texture

**Figure 4:**
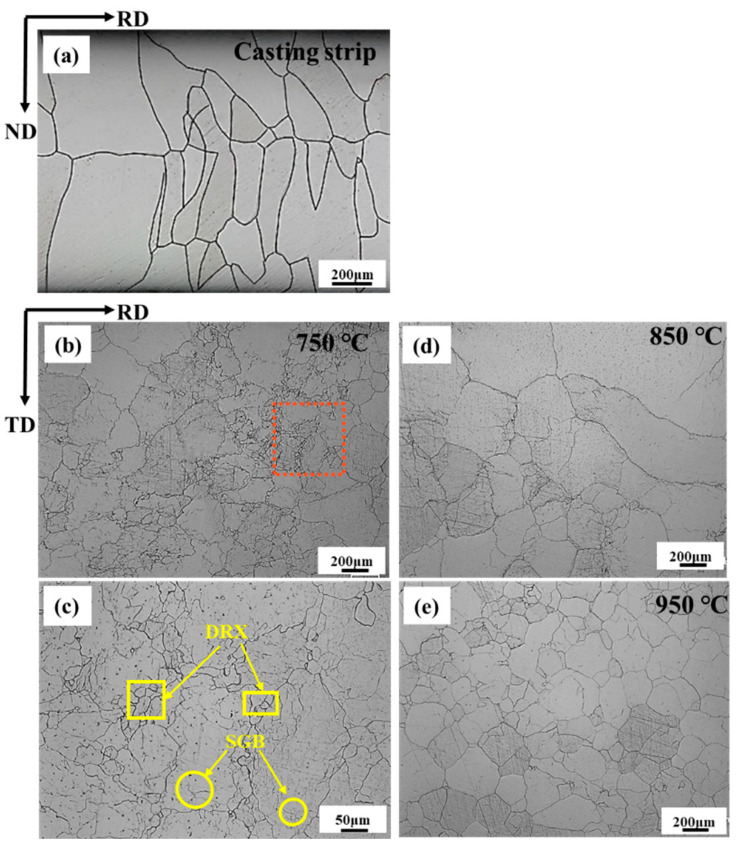
Microstructures of (**a**) the 2.43 wt.% Si steel as-cast strip and hot deformation strips obtained at different deformation temperatures of (**b**,**c**) 750 °C, (**d**) 850 °C, and (**e**) 950 °C with the rolling reduction of 30% and the strain rate of 0.1 s^−1^. (SGB stands for sub-grain boundary, DRX stands for dynamic recrystallization).

Figure 4 shows the microstructures of the electrical steel as-cast strip and hot-deformation strips obtained at different temperatures with the rolling reduction of 30% and the strain rate of 0.1 s^−1^. The electrical steel as-cast strip shows an average grain size of 296 μm in Figure 4a. Furthermore, coarse columnar crystals parallel to the solidification direction can be seen in the as-cast strip due to the high temperature gradient during the sub-rapid solidification of molten steel [29]. From Figure 4b,c, there are a large number of sub-grain boundaries within the grains, and recrystallized grains begin to nucleate at 750 °C. At 850 °C in Figure 4d, the high deformation temperature leads to the rapid accumulation of sub-grain boundaries. At this time, recrystallization and grain growth exist simultaneously. When the temperature rises to 950 °C, the grains are mainly recrystallized grains, as shown in Figure 4e. Grains grow and make the grain size distribution more uniform. The increase in deformation temperature improves the migration ability of grain boundary and promotes the nucleation and growth of DRX grains. 

**Figure 5:**
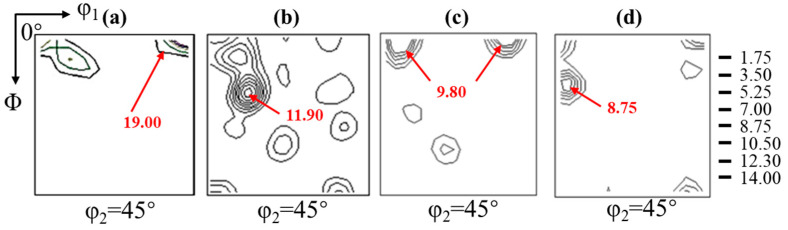
φ_2_ = 45° section of the orientation distribution function (ODF) of the 2.43 wt.% Si steel (**a**) strip cast and hot deformation strips obtained at different deformation temperatures of (**b**) 750 °C, (**c**) 850 °C, and (**d**) 950 °C with the rolling reduction of 30% and the strain rate of 0.1 s^−1^.

The φ_2_ = 45° section of the orientation distribution function (ODF) of a 2.43 wt.% Si steel strip cast and hot deformation at different temperatures is shown in Figure 5. The casting strip, as shown in Figure 5a, predominantly exhibits {001} fiber texture due to the development of columnar crystals during the sub-rapid solidification process. The hot deformation at 750 °C and the main orientation, including {001}, {117}, {112}, {110}, and {111}, are shown in Figure 5b. Combined with the microstructure, it can be observed that recrystallization initiates at this temperature with 0.1 s^−1^, leading to a diversified texture. The primary orientation of the hot-deformed plate at 850 °C is {001} in Figure 5c. This is mainly due to the growth of recrystallized and deformed grains with the increase in temperature, and the larger size of {001} grains inherited from the initial casting strip can merge with the surrounding small recrystallized grains. Completion of recrystallization occurs at 950 °C, resulting not only in {001} fiber texture but also α fiber texture ({113}), induced by external forces in Figure 5d.

**Figure 6:**
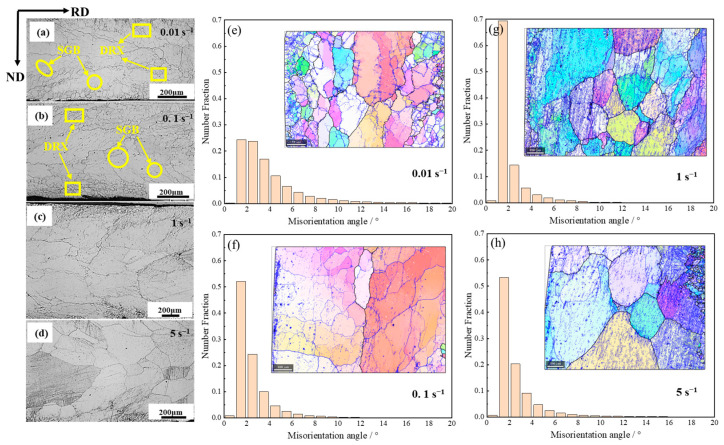
The microstructure and the misorientation angle distribution of the 2.43 wt.% Si steel strip obtained at different strain rates: (**a**,**e**) 0.01 s^−1^, (**b**,**f**) 0.1 s^−1^, (**c**,**g**) 1 s^−1^, (**d**,**h**) 5 s^−1^. The rolling temperature and rolling reduction are 950 °C and 30%, respectively. The black line is the high-angle grain boundary, and the blue line is the low-angle grain boundary.

The microstructures of test samples after deformation at different strain rates at 950 °C are shown in Figure 6. There are more recrystallized equiaxed grains in the samples. Figure 6a shows the recrystallized equiaxed grains with the size range of 40~100 μm in the sample under a strain rate of 0.01 s^−1^. This is because a large amount of recrystallization occurs at 750 °C. When the temperature rises to 950 °C, the recrystallized grains grow and merge smaller grains around them. With the strain rate increasing, the volume fraction of DRX and grain size decreases significantly. At 0.1 s^−1^ in Figure 6b, the recrystallization starts nucleation and grows from the surface layer to the central layer. At the strain rate of 1 s^−1^ in Figure 6c, the DRX occurs in the surface layer of the sample, and the middle is dominated by non-crystallized grains with sub-grain boundaries. When the strain rate rises to 5 s^−1^ in Figure 6d, the deformed and broken grains dominate the sample, and recrystallization does not occur in time due to the faster strain rate. 

Figure 6e–h show the diagram and misorientation angle distribution of grain boundary after hot deformation. The low-angle grain boundary misorientation is between 2° and 5°, the sub-grain boundary is less than 2°, and the high-angle grain boundary is more than 15°. With the increase in strain rate, sub-grain boundaries first increase and then decrease at 5 s^−1^, and low-angle grain boundaries first decrease and then increase. At 0.01 s^−1^, the sub-grain boundaries and low-angle boundaries are 24.41% and 51.2%. At 0.1 s^−1^, the sub-grain boundaries and low-angle boundaries increase to 52.12% and 38.91%. When the strain rate is 1 s^−1^, the sub-grain boundaries are 69.35% and the low-angle boundaries are 23.02%. When the strain rate is increased to 5 s^−1^, the sub-grain boundary decreases by 53.39% and the low-angle grain boundary increases to 34.14%. As the strain rate increases (from 0.01 s^−1^ to 0.1 s^−1^), the recrystallization rate and the high-angle boundary decreases, and dislocations more easily form the low-angle boundary [30]. So, the low-angle boundary does not have enough time to transition to the high-angle boundary. When the strain rate is 1 s^−1^, the sub-grain boundary ratio reaches the maximum due to a large number of substructures caused by high-density dislocation accumulation. At 5 s^−1^, the larger force is applied in a short time to cause grain deformation and refinement, and the dynamic recovery is mainly in the grain. So, the proportion of sub-grain boundaries decreases, while the proportion of low-angle grain boundaries increases.

**Figure 7:**
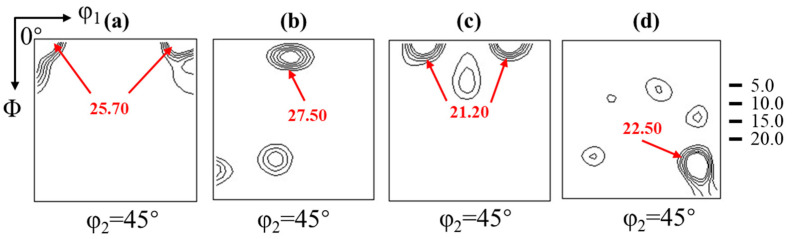
φ_2_ = 45° section of the orientation distribution function (ODF) of hot deformation strips obtained at different strain rates: (**a**) 0.01 s^−1^, (**b**) 0.1 s^−1^, (**c**) 1 s^−1^, (**d**) 5 s^−1^. The rolling temperature and rolling reduction are 950 °C and 30%, respectively.

Figure 7 shows the φ_2_ = 45° section of the orientation distribution function (ODF) of the 2.43 wt.% Si steel strip cast and hot deformation at different strain rates. At 0.01 s^−1^ in Figure 7a, it is dominated by the {001} orientation. At this time, recrystallization is obvious on both sides of the hot deformation plate, and the initial strip cast structure is inherited in the center layer. For the ODF of 0.1 s^−1^ hot deformation in Figure 7b, it can be found that the texture has {110} fiber texture and weaker α-fiber texture ({115}), which is because recrystallization starts at this time, {110} orientation is more preferentially nucleated, and there are sub-grain boundaries in grains, leading to the formation of {115} orientation. When the strain rate is 1 s^−1^ in Figure 7c, a large number of substructures caused by high-density dislocation accumulation are observed. Recrystallization does not occur obviously, and the λ-fiber texture changes to the {001} orientation. At the 5 s^−1^ in Figure 7d, the DRV is mainly in the grains, so the grain orientation is relatively diffuse.

### 3.3. Establishment of Constitutive Equation 

The constitutive equation is established according to the deformation conditions. And the parameter values are calculated.

At low stress (ασ < 0.8), Equation (2) was simplified as (3):

(3) $$\dot{\varepsilon }=A_{1}\sigma^{n*}$$

At high stresses (ασ > 1.2), Equation (2) was simplified as (4):

(4) $$\dot{\varepsilon }={\;A}_{2}\exp(\beta \sigma )$$

where the relationship between constants α, β, and n* is α = β/n* [31]. Taking the logarithm of each side of Equations (3) and (4), we obtain:

(5) $$\ln\sigma =-\ln A_{1}/a+\ln\dot{\varepsilon }/a$$

(6) $$\sigma =-\ln A_{2}/\beta +\ln\dot{\varepsilon }/\beta$$

According to the approximately linear relationship between ${\ln\sigma }_{p}-\ln\dot{\varepsilon }$ in Equation (5) and $\sigma_{p}-\ln\dot{\varepsilon }$ in Equation (6), we bring the data of the true stress–true strain curve into Equations (5) and (6), and take the reciprocal of its slope, averaged to obtain n* = 7.4593, β = 0.0714. By calculation, α = 0.00957.

Taking the logarithm of both sides of Equation (2), Equation (7) is deduced to show the relationship among the strain rate, temperature, and peak stress [32]:

(7) $$\ln\dot{\varepsilon }+\frac{Q}{\mathrm{RT}}=\mathrm{lnA}+n\ln\;[\sin h\left(\alpha \sigma_{p}\right)]$$

The variation of ln[sinh (α$\sigma_{p}$)] with $\ln\dot{\varepsilon }$ and 1/T was analyzed based on the true stress–true strain curves of 2.43 wt.% Si steel as-cast strip to acquire the strain rate sensitivity exponents of m = (1/*n*) and *Q*/(*n*R). 

**Figure 8:**
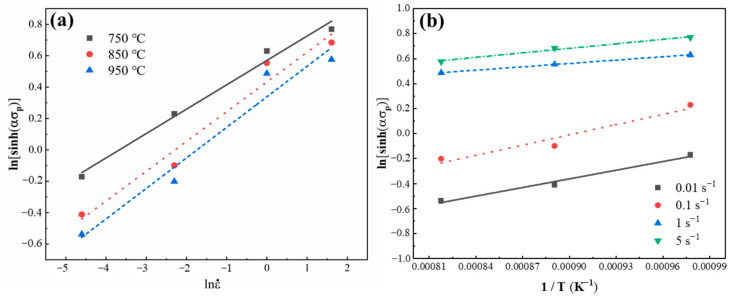
Variation of the hyperbolic sine function of peak true stress with (**a**) the strain rate at different deformation temperatures and with (**b**) the deformation temperature at different strain rates for the 2.43 wt.% Si steel as-cast strip.

As shown in Figure 8a, the plot shows the linear relationship between ln[sinh(ασ_p_)] and $\ln\dot{\varepsilon }$ at different hot deformation temperatures. The slope value at each deformation temperature is inversely proportional to *n*. Likewise, as can be seen in Figure 8b, the plots of ln[sinh(ασ_p_)] and 1/T are linearly proportional under a certain strain rate. It is obtained that *n* = 5.6195 and Q = 83,367.4676 (J/mol) for the studied 2.43 wt.% Si steel. Following this, the average value of A is 7.5366 × 10^3^. It has been reported that the thermal activation energies of Fe-6.5 wt.% Si, Fe-6.0 wt.% Si, Fe-5.5 wt.% Si, and Fe-3% Si non-oriented electrical steels produced by the traditional process are 422.162, 363.831, 310.425, and 289 kJ/mol, respectively [17]. It can be seen that the thermal activation energy of non-oriented electrical steel produced by twin-roll strip casting is lower than non-oriented electrical steel prepared by the traditional process, which can be attributed to the higher defect concentration, resulting from faster solidification rates in the casting strip.

According to the data obtained above, the Z and $\dot{\varepsilon }$ of experimental steel can be expressed as follows:

(8) $$Z=\dot{\;\varepsilon }\exp\left(\frac{83,367.4676}{\mathrm{RT}}\right)$$

(9) $$\dot{\varepsilon }\;=7.5366\;\times \;10^{3}{[\sin h\;(0.00957\sigma_{p})]}^{5.6195}\exp\;(\frac{-83,367.4676}{\mathrm{RT}})$$

**Figure 9:**
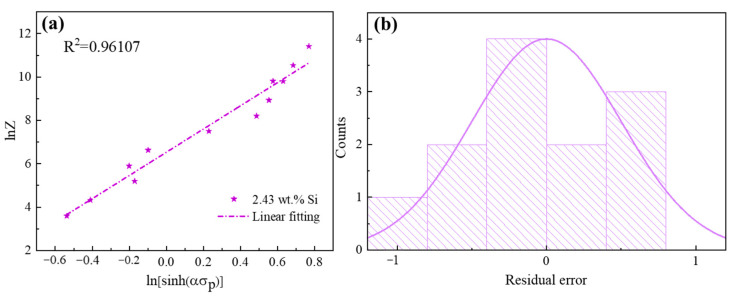
(**a**) Relationship between Z parameter and σ_p_ and (**b**) corresponding residual error following normal distribution for the 2.43 wt.% Si steel as-cast strip.

Figure 9 shows the plot of lnZ and ln[sinh(α$\sigma_{p}$)]. It can be found that the experimental data are close to the calculated results and demonstrate a fitting accuracy of 0.96107 for the data (Figure 9a). At the same time, the residual error is analyzed. It can be seen from Figure 9b that the residual error with the normal distribution demonstrates that the established constitutive equation is reasonable. Therefore, the relationship between peak stress and deformation conditions can be accurately described by the constitutive equation.

### 3.4. Dynamic Recrystallization Kinetics

**Figure 10:**
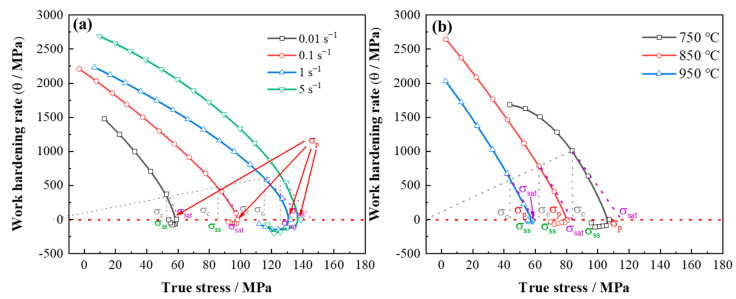
(**a**) The relationship between work hardening rate and true stress at different strain rates with the hot deformation of 950 °C, and (**b**) the relationship between work hardening rate and true stress at different temperatures with the strain rate of 0.1 s^−1^. σ_p_ is the peak stress, and σ_c_ is the critical stress.

The peak strain (ε_p_) corresponds to peak stress (σ_p_), which can be obtained directly from the stress–strain curve. The critical strain (ε_c_) corresponds to critical stress (σ_c_), where σ_c_ occurs at the inflection point of the curves (the point at which the second derivative of θ with respect to σ is equal to 0, i.e., $\partial^{2}\theta /\partial \sigma^{2}=0$). σ_c_ can be obtained from the work hardening rate–strain curve [33]. Figure 10 shows the typical θ-σ curves, from which the effect of hot rolling temperature and strain rate on work hardening rate can be found. The first intersection point of the θ-σ curve with θ = 0 is the peak stress. It can be clearly observed that the peak stress decreases with the increase in deformation temperature and increases with the increase in strain rate. The flow stress in Figure 10 decreases after the peak stress because the DRV and DRX behavior occurs. 

The relationship between strain and Z can be expressed as [34]:

ε = BZ^c^ (10)

where B and C are material constants. ε is peak strain (ε_p_) or critical strain (ε_c_). It can be found that lnε_p_ and lnε_c_ are linearly related to lnZ. The values of B and C can be obtained by averaging the intercepts or slopes of the lnε_p_-lnZ and lnε_c_-lnZ plots. Consequently, it can be calculated that the values of B and C are 0.056 and 0.066, respectively. Generally, ε_c_ has a linear relationship with ε_p_, i.e., ε_c_ = aε_p_, where a is the material constant. The hot deformation condition of 0.1 s^−1^ strain rate and 950 °C deformation temperature is selected for the calculation of a, because significant recrystallization occurs at this condition. It can be obtained that the value of a is 0.4863.

The kinetic model of DRX can be deduced from the Avrami equation [35,36], which can be expressed as follows:

(11) $$X_{\mathrm{DRX}}=1-\exp\left[\left(-k_{\mathrm{DRX}}\right)\cdot {\left(\frac{\varepsilon -\varepsilon_{c}}{\varepsilon_{p}}\right)}^{m_{\mathrm{DRX}}}\right](\varepsilon \;\leq \;\varepsilon_{c})$$

where k_DRX_ is the constant of the Avrami equation; m_DRX_ is the time constant, taking the natural logarithm of both sides of Equation (11). Taking the strain rate of 0.1 s^−1^ at different temperatures as an example, the relationship between $\ln[\ln\left(\frac{1}{1-X_{\mathrm{DRX}}}\right)]$ and $\ln(\frac{\varepsilon -\varepsilon_{c}}{\varepsilon_{p}})$ is known. Therefore, a linear regression analysis is applied to it, and m_DRX_ and k_DRX_ are obtained as 2.13934 and 0.54444, respectively.

Therefore, the DRX kinetics model of 2.43 wt.% Si steel strip cast is as follows:

(12) $$\left\{\begin{array}{c} X_{\mathrm{DRX}}=0,\;(\varepsilon \leq \varepsilon_{c})\\ X_{\mathrm{DRX}}=1-\exp\left[\left(-0.54444\right)\cdot {\left(\frac{\varepsilon -\varepsilon_{c}}{\varepsilon_{p}}\right)}^{2.13934}\right],\;(\varepsilon \;>\;\varepsilon_{c}) \end{array}\right.$$

**Figure 11:**
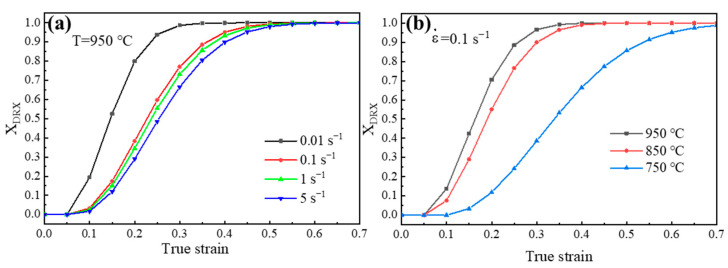
The relationship between DRX volume fraction and true strain at different (**a**) strain rates and (**b**) hot deformation temperatures.

According to the equation obtained above, the relationship between DRX volume fraction and true strain is shown in Figure 11. It can be found that when the hot deformation is at 950 °C and 0.01 s^−1^, the DRX of experimental steel can more easily reach the steady state. 

**Figure 12:**
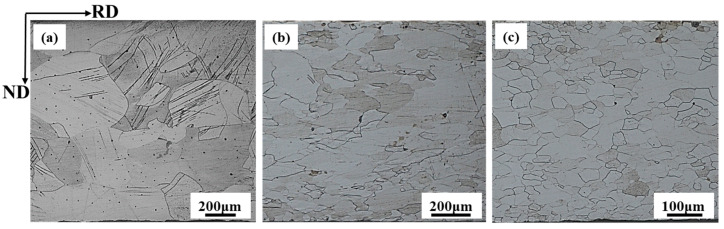
DRX of 2.43 wt.% Si steel strip prepared by rolling mill at different reduction rates: (**a**) 30%, (**b**) 50%, and (**c**) 70%. The strain rate is 0.1 s^−1^ and hot rolling temperature is 750 °C.

**Figure 13:**
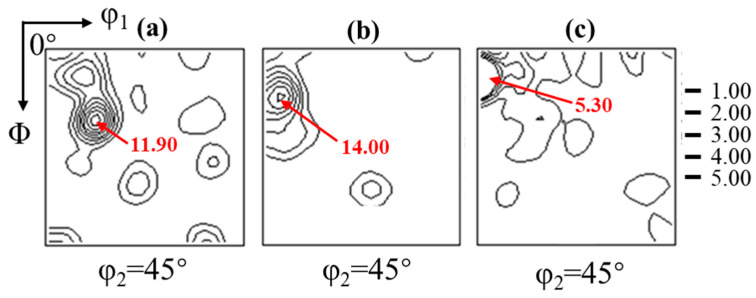
φ_2_ = 45° section of the orientation distribution function (ODF) for 2.43 wt.% Si experimental steel strips prepared by rolling mill at different reduction rates: (**a**) 30%, (**b**) 50%, and (**c**) 70%. The strain rate is 0.1 s^−1^ and hot rolling temperature is 750 °C.

The pilot rolling mill was used to conduct the hot rolling experiment on the 2.43 wt.% Si steel as-cast strip for verifying the DRX kinetics model. The optical microstructures of the samples obtained at different reduction rates are shown in Figure 12. According to the OM microstructure analysis of Figure 12a–c, it can be found that the DRX volume fraction of 2.43 wt.% Si steel strip tends to 100% when the strain is 70%, and the pattern of changes of the actual DRX volume fraction when pilot rolling is consistent with the model prediction. Figure 13 shows the ODF diagrams for 2.43 wt.% Si experimental steel strip prepared by rolling mill at different reduction rates. At the reduction rate of 30%, DRV and DRX happen simultaneously, resulting in complex textures. With the reduction rate increasing to 50%, the storage energy becomes larger, leading to an increase in the DRX volume fraction, and the texture of deformed grain stabilizes at α fiber texture with a new nucleation of {100} fiber texture. When the reduction rate is 70%, the increase in deformation leads to grains with serious external force and large energy storage, which provides more energy for DRX. The excessive deformation and DRX of the grains would refine the grains and make the texture become diffused, which has a negative impact on the magnetic properties. 

## 4. Conclusions

The hot deformation behavior of high-grade electrical steel produced by TRSC has not been well understood. In this article, the hot deformation behavior of 2.43 wt.% Si of silicon steel as-cast strip obtained via TRSC technology has been studied. The results could provide important guidelines for the parameter design of hot rolling during the actual strip casting process of silicon steel. The major conclusions have been summarized as follows:

(1) There were two types of true stress–strain curves during the hot deformation process of 2.43 wt.% Si steel as-cast strip. The DRX type of stress–strain curves occurred mainly at the strain rate from 0.01 s^−1^ to 0.1 s^−1^, while the DRV type of stress–strain curves mainly occurred at the strain rate from 1 s^−1^ to 5 s^−1^. The peak true stress rose with the increase in strain rate and the decrease in deformation temperature.
(2) With the increase in strain rate, the recrystallization volume fraction decreased, and recrystallization facilitated the formation of {001}, Goss, and rotated Goss texture.
(3) The deformation activation energy is 83.367 kJ/mol for the studied 2.43 wt.% Si electrical steel as-cast strip, which is lower than those of non-oriented electrical steels produced by the traditional process. A lower deformation activation energy is conducive to dynamic re-crystallization during the thermal deformation process. In addition, the constitutive equation for the studied electrical steel as-cast strip was established.
(4) The DRX kinetics model of 2.43 wt.% Si steel strip cast was established, and was verified by the pilot hot rolling experiment. The establishment of the dynamic recrystallization prediction model can provide guidelines for microstructure and texture control during the industrial production of TRSC high-grade steel strip.

## Data Availability

The data presented in this study are available on request from the corresponding author. The data are not publicly available due to technical or time limitations.
